# Combination Therapy of PEG-HM-3 and Methotrexate Retards Adjuvant-Induced Arthritis

**DOI:** 10.3390/ijms18071538

**Published:** 2017-07-21

**Authors:** Jingchao Hao, Xiaodong Wu, Sarra Setrerrahmane, Kun Qian, Yueying Hou, Liting Yu, Chenyu Lin, Qianqian Wu, Hanmei Xu

**Affiliations:** 1 The Engineering Research Centre of Peptide Drug Discovery and Development, China Pharmaceutical University, Nanjing 210009, China; jch_1180edu@126.com (J.H.); wuxiaodongcpu@126.com (X.W.); sarsetphar@gmail.com (S.S.); yult1992@126.com (L.Y.); linchenyu0427@gmail.com (C.L.); vicky577wqq@126.com (Q.W.); 2 School of Pharmaceutical Sciences & Yunnan Provincial Key Laboratory of Pharmacology for Natural Products, Kunming Medical University, Kunming 650500, China; 3 School of Life Science, Huzhou University, Huzhou 313000, China; qiankun@zjhu.edu.cn; 4 XiangYa School of Medicine, Central South University, Changsha 410013, China; hou250117626@126.com; 5 State Key Laboratory of Natural Medicines, Ministry of Education, China Pharmaceutical University, Nanjing 210009, China

**Keywords:** PEG-HM-3, peptide, methotrexate, rheumatoid arthritis, combination therapy, angiogenesis, inflammation

## Abstract

At present, the early phenomenon of inflammatory angiogenesis is rarely studied in Rheumatoid arthritis (RA). Previous research found that PEG-HM-3, an integrin inhibitor, possessed anti-angiogenesis and anti-rheumatic activity. In this study, the advantages of inhibiting angiogenesis and immune cell adhesion and migration, as well as the benefits of anti-arthritis effects, were evaluated using a combination of PEG-HM-3 and methotrexate (MTX). In vitro, spleen cell proliferation and the levels of tumor necrosis factor α (TNF-α) in macrophage supernatant were assessed. Hind paw edema, arthritis index, clinical score, body weight and immunohistochemistry (IHC) of the spleen, thymus, and joint cavity were evaluated in vivo in adjuvant-induced arthritis rats. Joints of the left hind paws were imaged by X-ray. The expression of the toll-like receptor 4 (TLR-4) protein was assessed in lipopolysaccharide (LPS)-induced synoviocytes. PEG-HM-3 combined with MTX significantly reduced primary and secondary swelling of the hind paws, the arthritis index, the clinical score and bone erosion. The results of IHC showed that the levels of interleukin-6 (IL-6) in spleens and the levels of TNF-α, CD31 (cluster of differentiation 31), and CD105 in the joint cavity were decreased. The body weight of rats was maintained during combination therapy. Ankle cavity integrity, and bone erosion and deformity were improved in combination treatment. The expression of TLR-4 was significantly reduced with combination treatment in rat synoviocytes. Co-suppression of both inflammation and angiogenesis in arthritis was achieved in this design with combination therapy. The activity of nuclear transcription factor (NF-κB) and the expression of inflammatory factors were down regulated via integrin α_v_β_3_ and TLR-4 signaling pathways. In the future, the application of this combination can be a candidate in early and mid-term RA therapy.

## 1. Introduction

Rheumatoid arthritis (RA) is an autoimmune disease characterized by a persistent chronic inflammation of synovium, leading to various degrees of cartilage destruction, bone erosion with subsequent joint deformity, and loss of joint function [1]. There are more than 5 million patients with RA in the world. Little is known about the initial activation of RA. Currently, it is believed that inflammation caused by antigen reaction leads to infiltration of macrophages which produce a large number of inflammatory factors in the synovium tissue, such as tumor necrosis factor α (TNF-α), interleukin-1 (IL-1), interleukin-6 (IL-6), and so on. Decades of studies have shown that synovial inflammatory angiogenesis is closely related to RA [2]. These cytokines can directly or indirectly promote angiogenesis through the vascular endothelial growth factor (VEGF) signaling pathway [3], resulting in synovial hyperplasia, bone destruction, and pannus. Some studies have reported that VEGF and its receptor antagonists can block the progression of neovascularization in RA [3,4,5,6,7,8]. So far, adjuvant-induced arthritis (AIA) and collagen-induced arthritis (CIA) are chronic arthritis animal models which can simulate the clinical symptoms of RA patients. The AIA model is induced by inactivated Bacillus Calmette-Guerin Vaccine (BCG) over a long-time and slow immune reaction [9]. The inflammatory reaction happens in the early stage of immune response followed by the formation of blood vessels.

Integrins are key targets for the treatments of inflammatory angiogenesis, which can not only regulate the expression levels of VEGF, but also affect the inflammatory trigger, bone cell development, migration, and bone resorption [10]. Some monoclonal antibodies, peptides, and small-molecule drugs can block the binding of integrins to their ligands [11,12]. 

Leukocytes migration into inflamed tissue can be suppressed by integrin antagonists. Natalizumab, a humanized monoclonal antibody, recognizes α_4_ integrin expressed on the surfaces of certain lymphocytes and is active in the treatment of multiple sclerosis (MS) and Crohn’s disease (CD) [13,14]. Natalizumab blocks the bindings of α_4_β_1_ integrin to vascular cell adhesion molecule 1 (VCAM-1) and α_4_β_7_ integrin to mucosal addressin cell adhesion molecule 1 (MadCAM-1) on endothelial cells in MS and CD [15,16]. The result of α_4_β_1_ integrin binding to fibronectin (FN) in the extracellular matrix may contribute to the pathological mechanism of RA [17]. Vedolizumab is the first humanized monoclonal antibody which can specifically target the α_4_β_7_ heterodimer and is approved by the US Food and Drug Administration for therapy of ulcerative colitis (UC) and CD [18]. Integrin α_4_ antibodies have also been used in animal models of autoimmune disease [19,20]. Several lines of evidence have demonstrated that osteoclast function can be regulated through blocking the α_v_β_3_ integrin-dependent signaling pathway in human bone diseases, including rheumatoid arthritis, osteoporosis, and periodontal disease [21]. A high level of integrin α_v_β_3_ is expressed on osteoclasts, which binds to a variety of extracellular matrix proteins including vitronectin, osteopontin, and bone sialoprotein. Evidence demonstrated that a number of signaling molecules, such as c-Src, Pyk2, c-Cbl, and p130Cas, are involved in the integrin α_v_β_3_ dependent signaling pathway. Integrin α_v_β_3_ was blocked to inhibit bone resorption. Therefore integrin α_v_β_3_ may be a novel target in RA therapy. However, until now, no integrin inhibitors have been successfully developed for RA therapy. 

We designed and synthesized HM-3 peptide, an angiogenesis inhibitor, which possesses ES-2 and integrin ligand RGD (Arg-Gly-Asp) sequences [22]. With modification of the N-terminus of HM-3 by methoxy-polyethyleneglycol-succinimidyl carbonate (mPEG-SC_20k_-, 20 kDa), we produced mPEG-SC_20k_-HM-3 (PEG-HM-3). It was revealed that PEG-HM-3 mainly targeted integrin α_v_β_3_ for the inhibition of tube formation, the migration of human umbilical vein endothelial cells (HUVECs), and angiogenesis in chick chorioallantoic membranes, in the previous studies [6,23]. 

It was demonstrated that PEG-HM-3 has an effective anti-rheumatic activity in AIA and CIA animal models with a dose of 10 mg·kg^−1^ in rats or 20 mg·kg^−1^ in mice [24]. In vitro, PEG-HM-3 significantly inhibited T cell proliferation and the expression levels of VEGF and TNF-α. However, the mechanism of PEG-HM-3 on arthritis antagonism is not clear. 

Methotrexate (MTX) is a classical medicine for RA treatment [4,5,25]; in fact, it can relieve patients’ symptoms but rarely cures the disease [7]. Indeed, many patients have a poor response to MTX, and they suffer from excruciating side effects from the application of long-term and high-dose MTX [26]. Possible bone toxicities and negative osteoblast activity, diffuse bone pain, and osteoporosis are accompanied by MTX treatment, which leads to anergy in patients. Moreover, MTX treatment alone dosen’t inhibit the expression of TNF-α and VEGF.

It is meaningful to reduce the dosages and untoward effects of drugs while improving efficacy. Therefore, a rational combination of drugs should be used in the treatment of RA. In recent years, TNF-α, IL-1, and IL-6 monoclonal antibodies have been widely used for RA therapy [5]. However, to increase the response rate (40–50%) and decrease side effects [27], it is not a good idea for single target therapy. Applications of drugs in combination with different targets are therefore used in RA, such as MTX in combination with a monoclonal antibody [3], a chemical compound [28], natural extraction products [29] or a peptide [30], and TNF-α monoclonal antibodies in combination with other antibodies [31]. However, these methods have no specific effect on the early stages of inflammatory angiogenesis. For the first time, we use an integrin α_v_β_3_ inhibitor in combination with MTX for the inhibition of inflammatory angiogenesis in the early stage of RA.

## 2. Results

### 2.1. Lymphoproliferative Responses and Anti-Inflammation Activity

**Figure 1:**
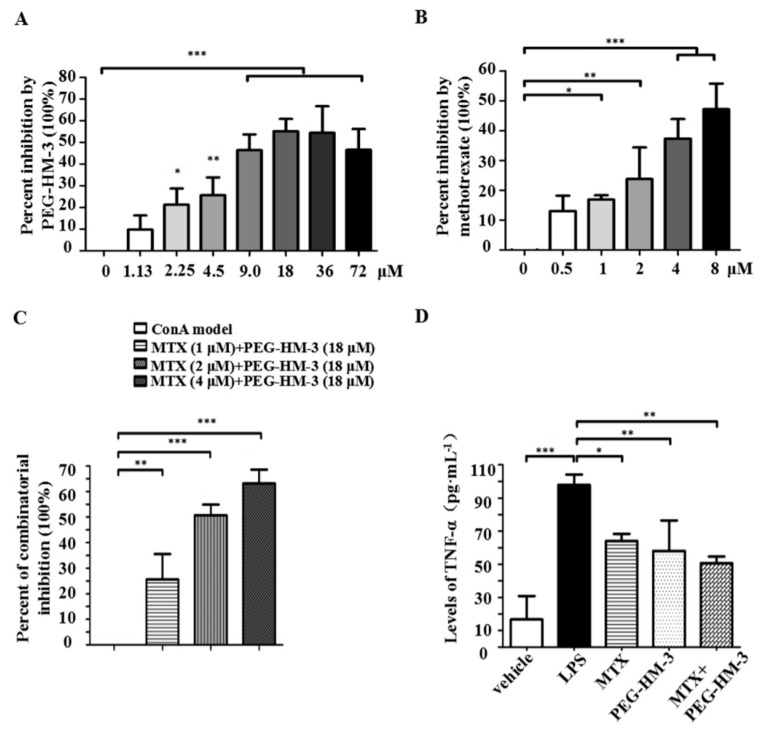
Effect of PEG-HM-3 alone or in combination with Methotrexate (MTX) on lymphoproliferative responses to mitogen ConA and anti-inflammation activity. (**A**) Inhibited proliferation with PEG-HM-3 (1.13–7.2 μM) in ConA (5 μg·mL^−1^)-induced splenocytes. (**B**) Inhibited proliferation with MTX (0.5–8 μM) in ConA (5 μg·mL^−1^)-induced splenocytes. (**C**) Dose-dependent inhibited proliferation with MTX in combination with fixed PEG-HM-3 (18 μM) in ConA (5 μg·mL^−1^)-induced splenocytes. (**D**) TNF-α levels in LPS (1 μg·mL^−1^)-induced RAW264.7 macrophage supernatants treated by MTX (1 μM), PEG-HM-3 (18 μM) or their combination. Values are means and standard error of the mean (SD) (*n* = 3 in (**A**,**B**); *n* = 4 in (**C**); *n* = 3 in (**D**)). The one-way ANOVA was used for group comparison. Versus ConA group or LPS group, * *p* < 0.05, ** *p* < 0.01 or *** *p* < 0.001.

To investigate the lymphoproliferative responses to the mitogen concanavalin A (ConA), splenocyte proliferation was tested. The ConA-induced proliferation of splenocytes was significantly inhibited by PEG-HM-3 in doses of 2.25–72 μM with a bell shape dose-efficacy relationship. The best inhibition rate (55.15%) was at 18 μM (*p* < 0.001) (Figure 1A). Proliferation was significantly inhibited in a dose-dependent manner by MTX alone in doses of 1–8 μM (*p* < 0.05) (Figure 1B). The inhibitory effects remained in the combination therapy groups. As shown in Figure 1C, the inhibitory effects were enhanced in combination therapy groups with an increasing dose of MTX plus a fixed dose of PEG-HM-3 (18 μM) (*p* < 0.01 or *p* < 0.001). 

To assess anti-inflammatory activity, TNF-α levels in macrophage supernatants were assessed (Figure 1D). Despite TNF-α levels being significantly decreased by MTX alone or by PEG-HM-3 alone, combination treatment of MTX and PEG-HM-3 showed the lowest TNF-α level (*p* < 0.01).

### 2.2. Efficacy in Adjuvant-Induced Arthritic Animals

**Figure 2:**
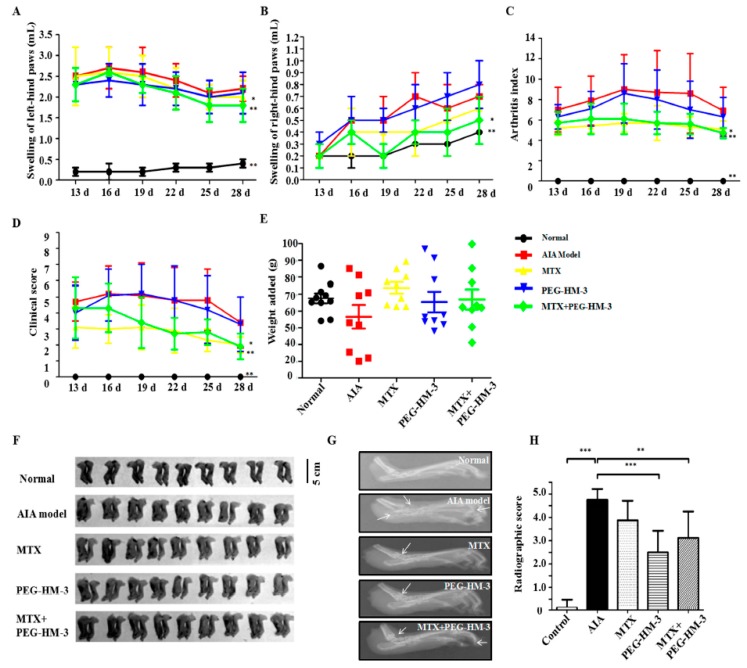
Curative effect of PEG-HM-3 alone or in combination with Methotrexate (MTX) on adjuvant-induced arthritis rats. All parameters were evaluated once every three days from the 13th day to the 28th day after disease onset (day 13, 16, 19, 22, 25, 28). (**A**) Swelling of the left-hind paws (mL); (**B**) Swelling of the right-hind paws (mL); (**C**) Arthritis index; (**D**) Clinical score; (**E**) Weight added (g) at the 28th day. MTX (1 mg·kg^−1^), PEG-HM-3 (10 mg·kg^−1^) and combination of MTX (1 mg·kg^−1^) and PEG-HM-3 (10 mg·kg^−1^) were used. Values are means and standard error of the mean (SD) ((**A**–**E**), *n* = 9 in each group); (**F**) Morphology of the left- and right-hinds paws in each group; (**G**) X-ray exhibition of the left-hind paws of rats at the end of the experiment; (**H**) The radiographic analysis of the left-hind paws (*n* = 8 in each group). Versus AIA model group. ** *p* < 0.01; *** *p* < 0.001.

To evaluate the combined effects of MTX and PEG-HM-3, AIA rats were tested in vivo during the clinical course. Primary inflammation occured in the left hind paws and started during the first day (Figure 2A). When used alone, PEG-HM-3 resulted in less swelling of the left hind paws than the control AIA model group and the MTX treatment alone group from the 13th day to the 22th day. At the end of the experiment, PEG-HM-3 treatment resulted in almost the same swelling as MTX treatment alone. Furthermore, from the 19th day to the 28th day, the combination of MTX and PEG-HM-3 resulted in the least swelling of the left hind paws (1.8 ± 0.4) (*p* < 0.01).

Secondary inflammation was produced in the right hind paws starting from the 13th day, which indicated a successful set up of the AIA model (Figure 2B). From the 19th day to the 28th day the swelling of right hind paws was significantly attenuated by the combination of MTX and PEG-HM-3 (*p* < 0.05). On the 28th day, the swelling of right hind paws was significant lower in the combination therapy group (0.5 ± 0.2) than in the MTX treatment alone group. 

Joint arthritis index was decreased in all therapeutic groups (Figure 2C). From the 13th day to the 28th day, the arthritis index was significantly attenuated by MTX treatment alone and by the combination of MTX and PEG-HM-3. At the 28th day, MTX combined with PEG-HM-3 resulted in a lower arthritis index (4.7 ± 0.5, *p* < 0.01) than that of MTX treatment alone (*p* < 0.05). However, no significant inhibitory effect was found by PEG-HM-3 treatment alone. 

The clinical score represents the severity of systemic inflammation caused by arthritis. The lowest clinical score was found in the MTX treatment group at all time points (Figure 2D). At the 28th day, MTX combined with PEG-HM-3 resulted in a lower clinical score (1.9 ± 0.8, *p* < 0.01) than MTX alone (2.3 ± 1.9, *p* < 0.05). However, no significance change was found in the PEG-HM-3 treatment group. 

Body weight generally decreases in clinical RA patients. Thus, the weight of arthritic rats was evaluated (Figure 2E). Compared with the AIA model group, body weight was significantly improved in all treatment groups. At the 28th day, a significant improvement in weight was found in the combination treatment group (274.3 ± 32.8, *p* < 0.01). The weights were also increased by MTX and by PEG-HM-3 treatment alone. 

As shown in Figure 2F, the morphology of left and right hind paws was observed in each group at the end of the experiment. Significant swelling was observed in left and right hind paws in the AIA model group. In all therapeatic groups, the lowest swelling was seen with the combination treatment of MTX and PEG-HM-3. Swelling of the hind paws was decreased in the MTX alone treatment group. The swelling was decreased by PEG-HM-3 alone, which was similar to the result of MTX alone. 

Severe irreversible deformations were observed in the joints of the AIA model group. Ankle joints suffered serious bone erosion, as being pointed out by white arrows on X-ray (Figure 2G). Compared with the AIA model group, less bone erosion and deformations were observed with the combination of MTX and PEG-HM-3. The joint space was narrowed with MTX or PEG-HM-3 treatment alone; however, the phenomenon of bone erosion was significantly reduced in the ankle joints. Deformations of the left hind paws were decreased in the MTX or PEG-HM-3 alone treatment group. Compared with the normal control rats, severe joint space narrowing and bone erosion were observed in the AIA model rats (4.75 ± 0.46) (Figure 2H). The lowest destruction of the joints was observed in the PEG-HM-3 group (2.5 ± 0.93, *p* < 0.001). It had a significant protective effect on joint space narrowing and bone erosion in the combination group (3.13 ± 1.13, *p* < 0.01). However, there was no significant difference between the MTX group and the AIA model group.

### 2.3. Histological Evaluation

**Figure 3:**
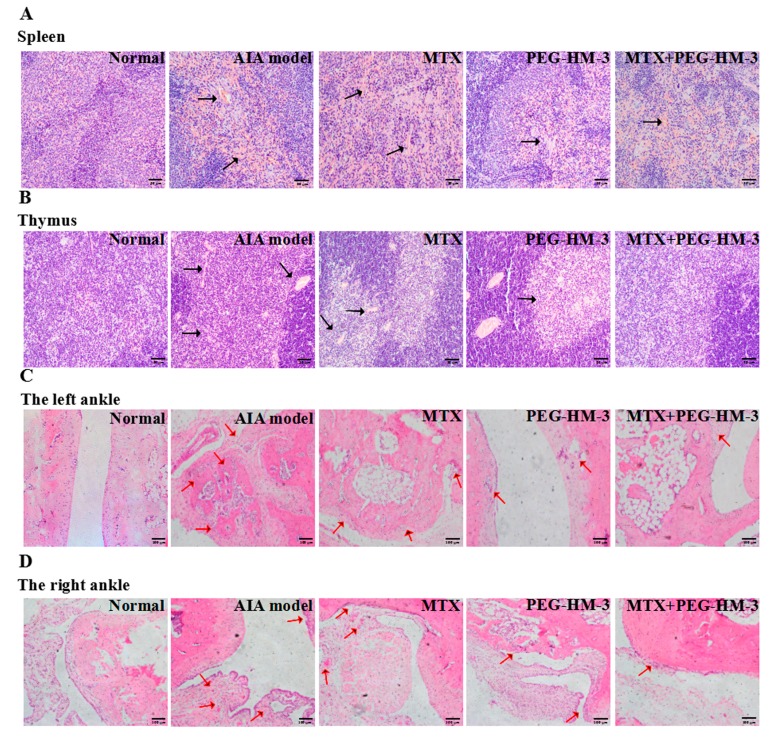
Histological staining in arthritic rats. (**A**) Histological staining of spleens; (**B**) Histological staining of thymus. Pathology areas were indicated with black arrows (*n* = 5–8 in each group, ×200 magnification); (**C**) Histological staining of the left-hind ankles; (**D**) Histological staining of the right-hind ankles. Images were observed by hematoxylin-eosin (HE) staining under inverted microscope. Pathological regions of synovial hyperplasia, pannus, inflammation and bone erosion were indicated by red arrows (*n* = 5–8 in each group, ×100 magnification).

To further investigate the efficacy of combination therapy in vivo, the spleen, thymus, and joint cavity were analyzed at a histological level. As shown in Figure 3A, there was no abnormal tissue in the negative control group (grade 0). Serious symptoms of increased cell proliferation, hyperemia, prominent germinalcenter (GC), and white pulp were seen in the spleens in the AIA model group (grade 3). Compared with the AIA model group, the combination of MTX and PEG-HM-3 resulted in decreased symptoms (grade 1). The pathology was also significantly decreased in the PEG-HM-3 treatment group (grade 1). Cell proliferation, marginal zone, and the appearance of prominent GC and white pulp in the spleen were reduced by MTX (grade 2). 

Compared with the control group, severe lymph follicular hyperplasia and thinning of the thymic cortex and medulla were obviously shown in the thymus of the AIA model group (Figure 3B). These symptoms were significantly alleviated by the combination of MTX and PEG-HM-3, in which organizational structure was restored to a certain extent. MTX alone resulted in slightly decreased lymph follicular hyperplasia in the thymic cortex and medulla, and less lymph follicular hyperplasia was found with PEG-HM-3 alone.

Arthritis of the hind paws was analyzed at the histological level (Figure 3, Table 1). The lowest values of pathological symptoms of synovitis, inflammation, and bone erosion were in the combination treatment group. Pannus in right-hind paws was also down-regulated by PEG-HM-3 treatment. Inflammation was reduced by MTX treatment alone in both of the hind paws. However, pannus was inhibited very little in MTX treatment.

### 2.4. Immunohistochemical Analysis

**Figure 4:**
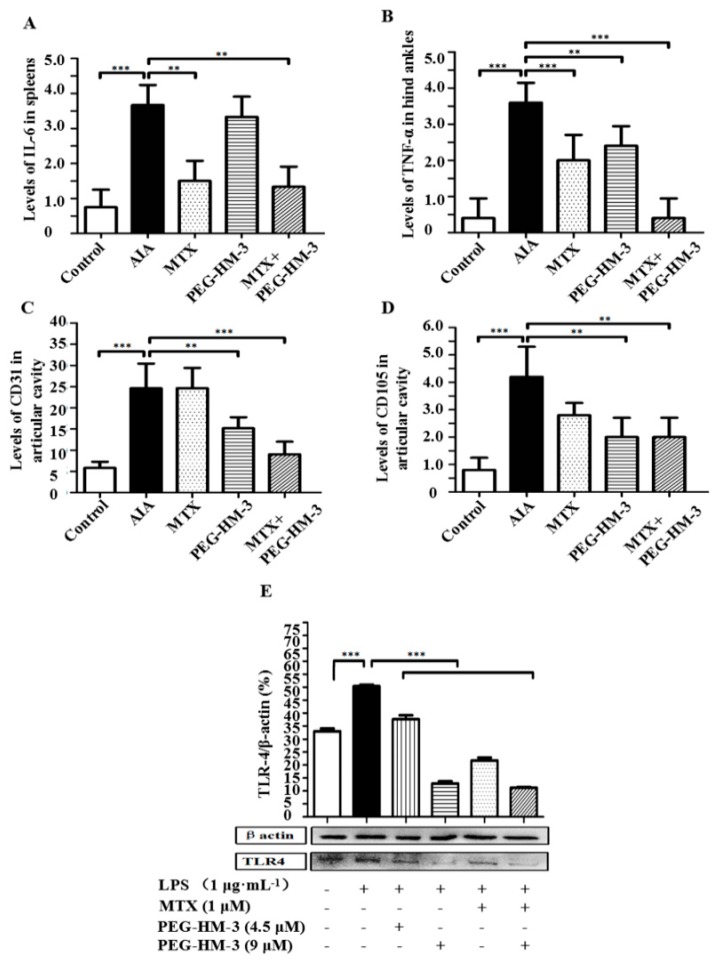
Immunohistochemical and western blot analysis of the levels of cytokines and proteins. (**A**) IL-6 expressions in spleens; (**B**) TNF-α expressions in hind ankles; (**C**) Levels of CD31 in joint cavity; (**D**) Levels of CD105 in joint cavity; (**C**,**D**) *n* = 5–8 in each group, ×200 magnification); (**E**) Western blot analysis of expressions of TLR-4 in synovial of rat (*n* = 3). The one-way ANOVA was used for group comparison. Versus LPS group, ** *p* < 0.01 or *** *p* < 0.001.

**Figure 5:**
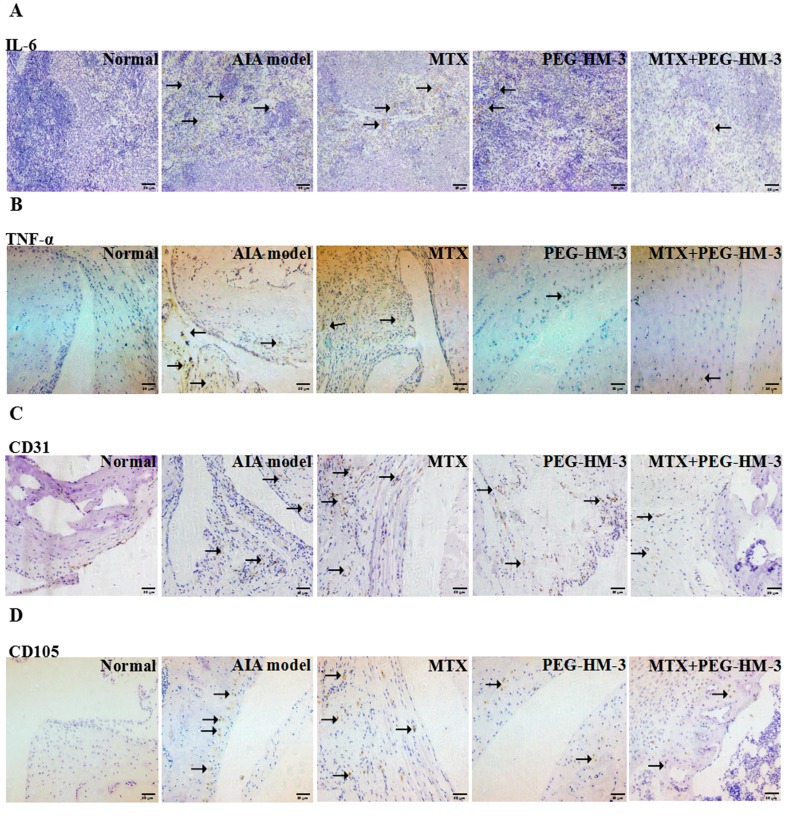
Immunohistochemical analysis in arthritic rats. (**A**) Expressions of IL-6 in spleens; (**B**) Expressions of TNF-α in hind ankles; (**C**) Expressions of CD31 in joint cavity; (**D**) Expressions of CD105 in joint cavity. The positive cells were stained brown and yellow and were indicated with black arrows. Values are means and standard error of the mean (SD) (**A**–**D**), *n* = 5–8 in each group, ×200 magnification).

The expression levels of IL-6 in the spleens were evaluated by Immunohistochemical (IHC) analysis. Low expression of IL-6 was found in the MTX treatment group (*p* < 0.01) (Figure 4A and Figure 5A). The combination of MTX and PEG-HM-3 significantly reduced the expression of IL-6 (1.33 ± 0.58, *p* < 0.01) compared with the MTX treatment group, but high expression of IL-6 was found in the PEG-HM-3 treatment group. The expression of TNF-α in the articular cavities was also investigated (Figure 4B and Figure 5B). The combination of MTX and PEG-HM-3 resulted in the lowest level (0.4 ± 0.55, *p* < 0.001). MTX or PEG-HM-3 treatment resulted in a weak inhibition of TNF-α in the joint cavity (*p* < 0.01 or *p* < 0.001). 

To assess the inhibitory effects on angiogenesis, microvessel density (MVD) was evaluated. The MVD was evaluated by the expression of CD31 in the joint cavity. As shown in Figure 4C and Figure 5C, CD31-positive fields were mainly adjacent to the synovial fibroblasts. It was interesting that small blood vessels were dyed by the CD31-positive antibody, which were surrounded by an annular monolayer of synovial fibroblasts. The lowest expression of CD31 was present in the combination treatment group (9.0 ± 3.0, *p* < 0.001). PEG-HM-3 treatment alone resulted in a significantly lower expression level than the AIA model group (15.2 ± 2.6, *p* < 0.01). However, MTX treatment had no effect on the expression of CD31. The CD105 positive cells were mainly distributed in the articular cartilage layer and synovial cells (Figure 4D and Figure 5D). The highest inhibitory effect on the expression of CD105 was achieved by combination treatment (2.0 ± 0.71, *p* < 0.01) and PEG-HM-3 treatment (2.0 ± 0.71, *p* < 0.01). However, MTX treatment showed no significant effect on the expression of CD105.

### 2.5. Efficacy in the Expression of TLR-4 Protein

Toll-like receptors (TLRs) are the front line of the immune response. The expression of TLR-4 protein was significantly down-regulated by PEG-HM-3 (9 μM) treatment alone or in combination with MTX (*p* < 0.001) (Figure 4E). Although the expression of TLR-4 protein was inhibited by MTX treatment, the inhibition between PEG-HM-3 (4.5 μM) and MTX was not significantly different. It is suggested that the activity of TLR-4 protein was down-regulated by PEG-HM-3 treatment through the TLR signaling pathway.

## 3. Discussion

**Figure 6:**
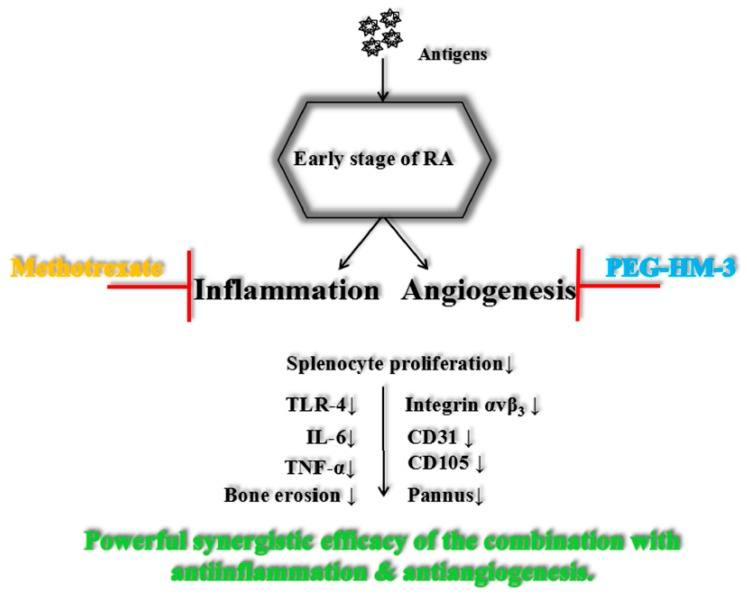
Schematic diagram of the combination therapy for early stage of RA. Black arrow represents a down regulation in the phenomena of splenocyte proliferation,bone erosion and pannus, expression of the proteins and contents of the cytokines.

Until now, no one integrin inhibitor for the treatment of RA has been applied, even a humanized monoclonal antibody to integrin α subunit, β subunit, or heterodimers. Previous studies have shown that PEG-HM-3 has a clear mechanism of inhibiting angiogenesis [23]. In this paper, the results illuminated treatment potentiality of PEG-HM-3 alone or in combination therapy for RA. We gained many insights into the development of multi-targeted peptides and the precise timing of their use in RA treatment. First of all, the peptide drug can enhance the safety and tolerability of treatments, which can slow down the adverse reactions caused by chemical drugs therapy. Secondly, it is very necessary to develop early treatments. Clinically, the time course is approximately 40 days from early symptoms to diagnosis. If an accurate combination therapy on inhibition of angiogenesis and primary and secondary arthritis is given during this course, patients can get a high response rate. It is earnestly suggested that the application of PEG-HM-3 in combination with MTX is most suitable for resisting inflammatory angiogenesis for early and mid-stage therapy. In the late stage of RA, combination therapy of PEG-HM-3 with other drugs may be based on relieving inflammation and pain. Thirdly, multiple target therapies are required in clinical, rather than a single target. Our research focused on improving the responses rate via multiple targeted therapy. IL-6 was decreased in the plasma of patients treated by MTX but TNF-α remained constant [32,33]. After adding PEG-HM-3, therapeutic targets were more efficiently and cooperatively blocked to inhibit the expression levels of VEGF, IL-6 [34,35,36,37], and TNF-α, through targeting integrin α_v_β_3_ and TLR-4 signaling pathways and the down-regulation of NF-κB activity (Figure 6). Therefore, we need to further explore the mechanism of PEG-HM-3 treatment alone in RA. 

Synovial cells and chondrocytes are the main targets, which are invaded and attacked by macrophages in the joint cavity. These processes are characterized by the cellular immune process, besides the effects of humoral immunity. The AIA model is mainly manifested through actions on the innate immune system, as seen by joint erosion and obvious and abnormal disorders in cellular immune regulation, such as the abnormal balance between Th1 and Th2 cell types. This pathological phenomenon is ideal for analyzing the production of inflammatory factors and pathological joint changes in early arthritis.

There is a need to study the effects of PEG-HM-3 on the CIA animals, although the CIA model is mainly characterized by acquired immunity (cellular and humoral immunity), and a complex pathogenesis. T cells and B cells play central roles in the CIA model, but the origin of immune activation and joint destruction is still unclear.

Direct and indirect evidence have shown that integrins were closely related to the pathogenesis of RA. Recruitment of immune cells to synovium is regulated by intercellular cell adhesion molecule-1 (ICAM-1) on endothelial cells and integrin αLβ_2_ on immune cells. Meanwhile, materials such as extracellular matrix (ECM) proteins, cytokines, adhesion molecules, and matrix-degrading enzymes are released by immune cells that can increase the expression of integrins. The expression of integrins again increases the expression of matrix metalloproteinases, which degrades ECM to small peptides and increases the expression of integrins, repeatedly, which promotes the development of RA [38]. RA synovial cells attach to cartilage fibronectin by integrins and other adhesion molecules. Integrin αv subunit is encoded by the *ITGAV* gene whose polymorphism is closely related to RA [39]. High expression of Integrin α_9_β_1_ was found in synovial fibroblasts and macrophages of RA patients [40]. Integrin α_10_β_1_ and α_11_β_1_ mediate cell adhesion to type-II collagen and affect the adhesion and migration of synovial fibroblasts and stem cells [41]. It was found that the loss of integrin α_2_β_1_ can inhibit inflammation and cartilage destruction in arthritic mice [42]. Integrin β_1_ may be another novel target to antagonize in RA.

In the adhesion pathway, different TLR signaling pathways (especially TLR-2, TLR-4) can activate integrin-dependent neutrophil adhesion and migration [43,44]. These TLRs can trigger downstream transcription factor kappa B (NF-κB) to transcribe and express inflammatory factors [45,46]. Compared with TLR-2, the function of TLR-4 was more closely related to RA [47,48]. The expression of TLR-4 in RA synovial tissue lining macrophages, fibroblasts, and sublining macrophages correlated with endogenous ligands [49,50]. In this paper, it was surprising that PEG-HM-3 could inhibit the expression of TLR-4. There may be crosstalk between TLR-4 and integrin α_v_β_3_ signaling pathways through NF-κB. Further research is needed to unravel the mechanism of this action.

To improve the therapeutic effects, the response rate, and security, an integrin α_v_β_3_ inhibitor (PEG-HM-3) was first combined with MTX for anti-inflammation and anti-angiogenesis arthritic therapy. It is suggested that integrin inhibitors can be used alone or in combination at specific pathological stages of RA, including early stage RA. Such combination therapy can be a candidate for early stage clinical RA therapy.

## 4. Materials and Methods

### 4.1. Ethics Statement

The protocol was approved by the Ethics Committee of China Pharmaceutical University (Permit Number: SYXK2012-0035, date of approval: 20 July 2012). The care and treatment of these animals were operated in accordance with the Provisions and General Recommendation of Chinese Experimental Animals Administration Legislation. The content and procedure of all animal experiments complied with the relevant laws and regulations, and the use of laboratory animals and management committee of IACUC (institutional animal care and use committee) regulations. There were no alternative methods in the experiments. Cessation of vital signs during the euthanasia of rats was confirmed by professionals.

### 4.2. Materials

HM-3 (sequence: IVRRADRAAVPGGGGRGD) was synthesized by GL Biochem Co., Ltd. (Shanghai, China). Methoxy-polyethyleneglycol-succinimidyl carbonate (mPEG-SC_20k_-) was purchased from Beijing Kaizheng Biotech Development Co., Ltd. (Beijing, China). mPEG-SC_20k_-HM-3 (PEG-HM-3) was prepared and purified with more than 98.5% purity.

### 4.3. Cell Culture and Stimulation Conditions

Healthy Balb/c mice were euthanized by CO_2_ asphyxiation and cervical dislocation. Spleens were collected and used as a source of conventional immune cells. The splenocytes were freshly isolated with a sterile 200 mesh cytoscreener. Red blood cells were lysed by red blood cell lysis buffer (Leagene, Beijing, China). Splenocytes (1.0 × 10^6^ cells·mL^−1^) were subsequently washed and suspended in Dulbecco’s Modified Eagle Medium (DMEM) (Gibco, Gaithersburg, MD, USA) containing 10% fetal bovine serum (FBS) (Biological Industries, Beit HaEmek, Israel), and were cultured at 37 °C in 96-well plates in 5% CO_2_ for 24 h. Vehicle or ConA (5 μg·mL^−1^) were used as a negative or positive control. Splenocytes were treated with different doses of PEG-HM-3 (1.13–72.0 μM) in the presence or absence of MTX (1, 2, 4 μM) (Hengrui medicine Co Ltd., Lianyungang, China) for 48 h. 

RAW264.7 cells were maintained in RPMI 1640 culture medium (Gibco, USA) with 10% FBS until the logarithmic growth phase. RAW264.7 cells were differentiated into macrophages by treatment with 1 μg·mL^−1^ lipopolysaccharide (LPS) (Sigma, St. Louis, MO, USA) for 48 h [51]. Adherent macrophages were cultured at 37 °C with 5% CO_2_. Macrophages were treated with PEG-HM-3 (18 μM) in the presence or absence of MTX (1 μM) for 48 h.

Synovial tissue was obtained from the hind paws of rats, and synovium was mechanically dispersed into small pieces and plated in a 6-well plate (Thermo Fisher Scientific, Waltham, MA, USA) in DMEM supplemented with 10% FBS, 100 U·mL^−1^ penicillin, 100 μg·mL^−1^ streptomycin (Amresco, Solon, OH, USA). Cultures were kept at 37 °C in a 5% CO_2_ incubator. The culture medium was changed every 2 days. At 80% confluence, cells were serially subcultured with 0.25% trypsin-ethylene diamine tetraacetic acid solution and were then subjected to experiments. Synoviocytes were induced by LPS (1 μg·mL^−1^) in 6-well culture plates for 48 h. Then, cells were treated with PEG-HM-3 (4.5 or 9.0 μM) alone or in combination with MTX (1 μM) for 48 h.

### 4.4. MTT and ELISA Analysis

The lymphoproliferative responses were evaluated by an MTT method [52]. 20 μL MTT (5 mg·mL^−1^) was added to a 96-well plate and incubated at 37 °C for 4 h. After washing off supernatants, 150 μL DMSO was added and the plate was shaken for 5 min. The optical density (OD) value was measured under 570 nm and 630 nm wavelength under an enzyme marking instrument (Thermo Fisher Scientific, USA).

∆OD = OD_570 nm_ − OD_630 nm_ (1)

Inhibition rate (100%) = (1 − ∆OD_test_/∆OD_ConA_) × 100% (2)

Macrophage supernatants were analyzed by an enzyme linked immunosorbent assay (ELISA) kit (Donglin Technology Development Co. Ltd., Wuxi, China) under 450 nm in a multifunctional enzyme marking instrument (Thermo Fisher Scientific, USA), following the protocol of the kit. The regression equation of the standard curve for TNF-α content measurement was *y* = 0.4268*x* + 0.0692 (*R*^2^ = 0.9997). The content of TNF-α was calculated on the basis of the detected OD value. 

### 4.5. Animals and Experiment Design

Male Sprague Dawley (SD) rats (6 to 8 weeks) were obtained from the BK Experimental Animal Center (Shanghai, China) and were used for induction of the AIA model. All animals were housed in a controlled environment (22 ± 2 °C; 12 h light-dark cycle) under specific pathogen-free conditions with water and food provided adlibitum. Rat adjuvant arthritis was induced as recommended [53]. The AIA rat was induced by intradermal injection in the left hind paw with 0.1 mL Complete Freund’s adjuvant (*M. tuberculosis* H37RA, 10 mg·mL^−1^, Chondrex, Redmond, WA, USA) on the first day. After immunization, rats developed arthritis symptoms on the 13th day. The experiment included healthy animals (normal), arthritic animals treated with saline (AIA model), arthritic animals treated with MTX (1.0 mg·kg^−1^), PEG-HM-3 (10 mg·kg^−1^), or the combination of MTX (1.0 mg·kg^−1^) and PEG-HM-3 (10 mg·kg^−1^). The combination group received 10 mg·kg^−1^ PEG-HM-3 subcutaneously once every two days and 1.0 mg·kg^−1^ MTX subcutaneously once every 5 days during the experiment. The period of treatment was 2 weeks. There were nine animals in each group. All experiments had been performed in compliance with the Guide for the Care and Use of Laboratory Animals. After the animals had been sacrificed under sodium pentobarbital anesthesia (op.), tissues (spleens, thymus, and left hind and right hind paws) were collected.

### 4.6. Assessment of Arthritis Symptom

From the 13th day, the swelling of the left and right hind paws were measured once every 3 days. Histologic sections were evaluated by two independent observers without any knowledge of experimental groups. The swelling of paws was determined by water volume (the displacement method):

swelling (mL) = paw thickness (mL) _tested value_ − paw thickness (mL) _original value_ (3)

The arthritic index was assessed once every 3 days from the 13th day. Arthritis severity was scored by grading each paw from 0 to 4 based on erythema, swelling, and deformity of the joint: 0 = no erythema or swelling; 1 = slight erythema or swelling of one of the toes or fingers; 2 = erythema and swelling of more than one toe or finger; 3 = erythema and swelling of the ankle or wrist; 4 = complete erythema and swelling of toes or fingers and ankle or wrist, and the inability to bend the ankle or wrist. All four paws were scored and the maximum possible score per rat was 16. The clinical score assessment for the systematic inflammation lies between 0 and 8 for each animal (sum of 1 = nose, 1 = left ear, 1 = right ear, 1 = left paw, 1 = right paw, 1 = left hind paw, 1 = right hind paw, 1 = tail). Body weight of the rat was measured every 3 days with a precision of 0.1 g. At the end of the experiment, rats were dissected and left hind paws imaged by X-ray machines. X-rays were scored for the ankles and tarsus joints from 0 to 5 according to the grade of destruction: 0 = normal; 1 = demineralization; 2 = narrowing, 50% of joint space; 3 = narrowing, 50% of joint space and joint erosion; 4 = loss of joint space; 5 = complete joint destruction [54]. The X-rays were assessed by unwitting professionals.

### 4.7. Histopathological Analysis

Spleens and thymus were fixed in 10% neutral formaldehyde. The left and right hind paws were collected and decalcified by 5.0% ethylene diamine tetraacetic acid (EDTA) in 10% formaldehyde at room temperature. Liquid was changed twice a week for eight weeks. The tissues were embedded in paraffin, and serial sections (5 μm) were cut and mounted on glass slides and stained with hematoxylin-eosin (HE) for examination under a microscope. The sections were examined under the inverted microscope (Olympus, Tokyo, Japan). Histopathological changes of the spleen were graded from 0 to 3: grade 0, normal spleen; grade 1, mild proliferation of white pulp; grade 2, moderate proliferation of white pulp; grade 3, severe proliferation of white pulp and prominent germinal center [55]. Lymphoid proliferation was evaluated in the thymus [56]. 

A histological assessment of arthritis severity was made by blinded evaluation of the HE-stained ankle joint cavity in accordance with a scoring system: 0 = normal; 1 = mild hyperplasia of the synovial lining layer; 2 to 4 = synovial lining hyperplasia and pannus formation. Inflammation was scored as the following: 0 = normal; 1 = mild inflammation (1 aggregate or few leukocyte infiltration); 2 = moderate inflammation (≥2 leukocyte aggregates); 3 = marked inflammation (large leukocyte aggregates and significant leukocyte infiltration). Bone erosion was scored as the following: 0 = normal; 1 = minimal (1–2 small, shallow sites); 2 = mild (1–4 sites of medium size and depth); 3 = moderate (5 sites partially extending through the cortical bone); 4 = marked (multiple foci partially or completely extending through the cortical bone); 5 = extensive (cortical penetration at 25% of bone length). Two independent observers evaluated the slides with no knowledge of the experimental groups.

The expression levels of IL-6 in spleens and TNF-α, CD31, CD105 in ankle joints were analyzed. The sections were mounted on glass slides and fixed in acetone, and then preincubated with rabbit anti-rat serum for 20 min to reduce nonspecific staining at the room temperature. This was followed by incubation with 3% hydrogen peroxidase for 10 min to inhibit endogenous peroxidases. The slides were sequentially incubated with specific anti-rat antibodies against IL-6, TNF-α, CD31, or CD105 (Santa Cruz, CA, USA) overnight at 4 °C. After primary layer antibody incubation, the slides were incubated with appropriate second layer antibodies for 30 min at 37 °C. The sections were washed with PBS and stained with 3,3-diaminobenzidine (DAB) for 10 min. Then, they were stained with hematoxylin, dehydrated with 95% ethanol, and sealed with neutral resin for examination under a microscope. The brown positive cells in the sections were identified and counted by two investigators with no knowledge of the experimental groups under an inverted microscope. The numbers of positive cells in the AIA model group was represented.

### 4.8. Western Blot Assay of Protein Expressions

Western blot assay was performed to verify the expression of the key protein TLR-4. The synoviocytes were resuspended in 120 μL of mammalian protein extraction reagent (Shanghai Generay Biotech Co., Ltd., Shanghai, China). Concentrated protein was separated on a 12% SDS-polyacrylamide gel (SDS-PAGE), and the protein bands were transferred to a 0.22 μm aperture polyvinylidene fluoride (PVDF) membrane. Then the PVDF membrane was incubated with a 1:1000 dilution of TLR-4 or β-actin antibodies (Cell Signaling Technology, Danvers, MA, USA). Next, the membrane was incubated with a 1:2000 dilution of secondary antibodies and the bands were visualized with an electro-chemi-luminescence (ECL) reagent.

### 4.9. Statistical Analysis

The data were analyzed by SPSS software (version 17.0; SPSS Inc., Chicago, IL, USA) and GraphPad software (version 5.01; GraphPad Inc., La Jolla, CA, USA). The results were expressed as mean ± SD. The analysis of variance (ANOVA), one-way ANOVA, and the independent *t*-test were used for group comparison. For all statistical analyses, a *p* value of less than 0.05 (2-tailed) was used to test for statistical significance. All reported probabilities are two tailed.

## Figures and Tables

**Table 1:** Histological inflammation score in the joint cavity in arthritis rats.

Left Hind Paws	Normal	AIA Model	MTX	PEG-HM-3	MTX + PEG-HM-3
Synovitis	0.00 ± 0.00 ***	3.00 ± 0.82	1.33 ± 0.58 **	1.33 ± 0.58 **	1.00 ± 0.00 ***
Pannus	0.00 ± 0.00 ***	3.17 ± 0.29	2.00 ± 0.82	1.67 ± 0.58 *	1.33 ± 0.58 **
Inflammation	0.00 ± 0.00 ***	2.67 ± 0.58	1.33 ± 0.58 *	1.33 ± 0.58 *	1.00 ± 0.00 **
Bone erosion	0.00 ± 0.00 ***	4.33 ± 0.58	1.33 ± 0.58 ***	1.33 ± 0.58 ***	1.00 ± 0.00 **
**Right Hind Paws**	**Normal**	**AIA Model**	**MTX**	**PEG-HM-3**	**MTX + PEG-HM-3**
Synovitis	0.00 ± 0.00 ***	3.33 ± 0.58	1.67 ± 0.58 **	1.33 ± 0.58 ***	1.00 ± 0.00 ***
Pannus	0.00 ± 0.00 ***	3.67 ± 0.58	1.67 ± 0.58 **	1.17 ± 0.29 ***	1.00 ± 0.00 ***
Inflammation	0.00 ± 0.00 ***	2.67 ± 0.58	1.00 ± 0.00 ***	1.33 ± 0.58 **	1.00 ± 0.00 ***
Bone erosion	0.00 ± 0.00 ***	3.33 ± 0.58	1.67 ± 0.58 **	1.33 ± 0.58 ***	1.33 ± 0.58 ***

vs. the AIA model, * *p* < 0.05; ** *p* < 0.01 or *** *p* < 0.001.

## Abbreviations

AIA	antigen-induced arthritis
CD	Crohn’s disease
CD	cluster of differentiation
CFA	complete Freund’s adjuvant
CIA	type II collagen-induced arthritis
ConA	concanavalin
DMARDs	disease-modifying anti-rheumatic drugs
EDTA	ethylene diamine tetraacetic Acid
ELISA	enzyme linked immunosorbent assay
HPLC	high-performance liquid chromatography
IL	interleukin
LPS	lipopolysaccharide
MTT	methylthiazolyldiphenyl-tetrazolium bromide
MTX	methotrexate
mPEG-SC_20k_-	methoxy-polyethylene glycol-succinimidyl carbonate
PEG	polyethylene glycol
PEG-HM-3	mPEG-SC_20k_-HM-3
RA	rheumatoid arthritis
RGD	Arg-Gly-Asp
SD	Sprague Dawley
TLRs	toll-like receptors
TNF	tumor necrosis factor
VEGF	vascular endothelial cell growth factor
